# Increased proviral load in HTLV-1-infected patients with rheumatoid arthritis or connective tissue disease

**DOI:** 10.1186/1742-4690-2-4

**Published:** 2005-02-01

**Authors:** Maria Yakova, Agnès Lézin, Fabienne Dantin, Gisèle Lagathu, Stéphane Olindo, Georges Jean-Baptiste, Serge Arfi, Raymond Césaire

**Affiliations:** 1 INSERM UMR433, antenne du Centre hospitalier universitaire de Fort-de-France, Martinique; 2 Service de Médecine interne et Rhumatologie, Centre hospitalier universitaire de Fort-de-France, Martinique; 3 Laboratoire de Virologie-Immunologie, Centre hospitalier universitaire de Fort-de-France, Martinique; 4 Service de Neurologie, Centre hospitalier universitaire de Fort-de-France, Martinique

## Abstract

**Background:**

Human T-lymphotropic virus type 1 (HTLV-1) proviral load is related to the development of HTLV-1-associated myelopathy/tropical spastic paraparesis (HAM/TSP) and has also been shown to be elevated in the peripheral blood in HTLV-1-infected patients with uveitis or alveolitis. Increased proliferation of HTLV-1-infected cells in, or migration of such cells into, the central nervous system is also seen in HAM/TSP. In the present study, we evaluated the proviral load in a cohort of HTLV-1-infected patients with arthritic conditions.

**Results:**

HTLV-1 proviral load in the peripheral blood from 12 patients with RA and 6 patients with connective tissue disease was significantly higher than that in matched asymptomatic HTLV-1 carriers, but similar to that in matched HAM/TSP controls. HAM/TSP was seen in one-third of the HTLV-1-infected patients with RA or connective tissue disease, but did not account for the higher proviral load compared to the asymptomatic carrier group. The proviral load was increased in the synovial fluid and tissue from an HTLV-1-infected patient with RA, the values suggesting that the majority of infiltrated cells were HTLV-1-infected. In the peripheral blood from HTLV-1-infected patients with RA or connective tissue disease, HTLV-1 proviral load correlated with the percentages of memory CD4+ T cells and activated T cells, and these percentages were shown to be markedly higher in the synovial fluid than in the peripheral blood in an HTLV-1-infected patient with RA.

**Conclusions:**

These biological findings are consistent with a role of the retrovirus in the development of arthritis in HTLV-1-infected patients. A high level of HTLV-1-infected lymphocytes in the peripheral blood and their accumulation in situ might play a central role in the pathogenesis of HTLV-1-associated inflammatory disorders. Alternatively, the autoimmune arthritis, its etiological factors or treatments might secondarily enhance HTLV-1 proviral load.

## Background

Human T-lymphotropic virus type 1 (HTLV-1) is endemic in southern Japan, intertropical Africa, Melanesia, Latin America, and the Caribbean basin [1]. HTLV-1 is the etiological agent of adult T-cell leukemia [2] and HTLV-1-associated myelopathy/tropical spastic paraparesis (HAM/TSP), an inflammatory disease of the central nervous system [3,4], and has also been implicated in several other inflammatory disorders, such as polymyositis [5], uveitis [6], Sjögren's syndrome [7], alveolitis [8], and infective dermatitis [9].

The possibility that HTLV-1 may cause joint disease was initially raised by reports of arthralgia and polyarthritis in patients with adult T-cell leukemia [10,11]. Polyarthritis has also been observed in some patients with HAM/TSP [12]. Nishioka et al. [13] described the association of a polyarthritis syndrome with HTLV-1 infection in the absence of clinical ATL or HAM/TSP, and proposed the term HTLV-1-associated arthritis (HAA). Cases of HTLV-1-infected patients with mixed connective tissue disease have also been described [14], although an association between HTLV-1 infection and systemic lupus erythematosus has not been established [15]. Apart from the possibility of neurological signs, the clinical features of HAA are similar to those of idiopathic rheumatoid arthritis (RA) [16-18]. Epidemiological studies have demonstrated that HTLV-1 seropositivity is a risk factor for RA in Japan [19,20], but a recent study conducted in South Africa, another HTLV-1 endemic area, failed to detect any association between HTLV-1 and RA [21]. This discrepancy might be due to differences in genetic background, although the possibility cannot be excluded that HAA in Japan results from the coincidental coexistence of two relatively common diseases. Interestingly, a recent prospective study demonstrated an increased incidence of arthritis in cohorts of former US blood donors infected with HTLV-1 or HTLV-2 [22].

Several findings support the hypothesis of an etiopathogenic role for HTLV-1 in HAA: ATL-like T lymphocytes have been identified in the synovial fluid and synovial tissue [17,18,23]; high titers of IgM antibodies against HTLV-1 have been found in the synovial fluid [23]; HTLV-1 proviral DNA has been detected in synovial fluid cells and synovial tissue cells [23], cultured adherent synovial stromal cells [24], and synovial macrophage cells [25]; and Tax mRNA and protein have been detected in synovial stromal cells [26]. HTLV-1 tropism for synovial cells has been confirmed in vitro [27]. Moreover, mice transgenic for Tax develop an inflammatory arthropathy resembling RA in humans [28]. The development and progression of RA is dependent on the migration of T lymphocytes into the synovial compartment [29,30]. Similarly, the tissue damage in HAM/TSP is thought to be caused by T cells that have infiltrated the central nervous system [31,32]. T lymphocytes, especially CD4^+ ^T cells, are the main target of HTLV-1 in vivo and carry the majority of the HTLV-1 proviral load [33]. The HTLV-1 proviral load in peripheral blood mononuclear cells (PBMCs) is higher in patients with HAM/TSP than in asymptomatic HTLV-1 carriers [34] and the equilibrium set point of the proviral load is suspected to determine the development of the disease [35].

We postulated that HTLV-1 proviral load might also influence the initiation and course of HAA, and measured this marker in PBMCs from a previously described cohort [16] of HTLV-1-infected patients with RA and in a group of HTLV-1-infected patients with connective tissue disease.

## Results

**Figure 1:**
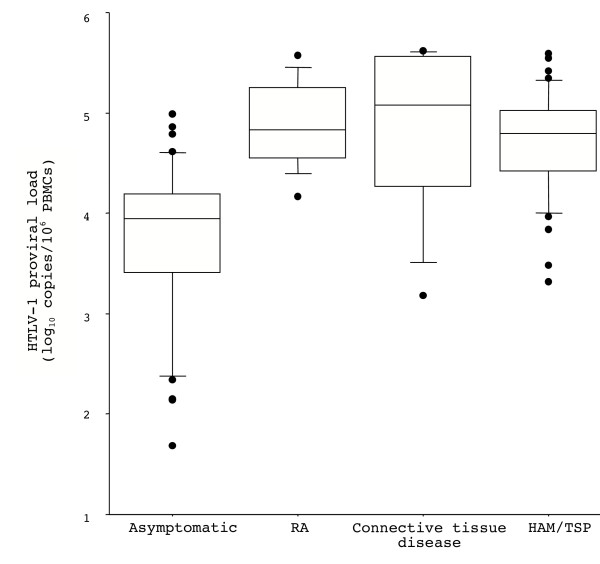
**HTLV-1 proviral load in the peripheral blood from HTLV-1-infected patients with RA or connective tissue disease and from HAM/TSP or asymptomatic controls. **The 10^th ^and 90^th ^percentiles are shown as the lower and upper horizontal bars on the vertical line, while the 25^th ^and 75^th ^percentiles are shown as the lower and upper edges of the box; the median is shown within the box. The results shown as dots fall outside the 10^th ^and 90^th ^percentiles.

The HTLV-1 proviral load was measured in the peripheral blood of HTLV-1-infected patients with RA or connective tissue disease and in matched asymptomatic and HAM/TSP controls (Figure 1). The number of copies of HTLV-1 proviral DNA per 10^6 ^PBMCs ranged from 14,600 to 373,000 in the patients with RA (Table 1) and from 1,500 to 411,200 in patients with connective tissue disease (Table 2), the corresponding ranges in the HTLV-1 asymptomatic carriers and in patients with HAM/TSP being 50 to 97,700 and 2,100 to 392,000, respectively. The mean ± SD and median proviral loads were 133,800 ± 134,600 and 75,800 in the HTLV-1-infected patients with RA or connective tissue disease combined, the values for the RA subgroup being 114,400 ± 112,200 and 67,400 and those for the connective tissue disease subgroup 172,500 ± 176,700 and 120,800, while the corresponding values in the asymptomatic carriers were 18,800 ± 26,400 and 10,100 and those in patients with HAM/TSP 86,800 ± 90,600 and 62,400. The HTLV-1 proviral load was significantly higher in the HTLV-1-infected group with RA or connective tissue disease than in the matched asymptomatic HTLV-1 carriers (*P *= 0.0012 in Wilcoxon's test, *P *= 0.0002 in the paired *t*-test after log-transformation), and the difference remained significant when the analysis focused on the RA subgroup (*P *= 0.0022 in Wilcoxon's test, *P *= 0.0002 in the paired *t*-test). No differences were observed between the HTLV-1-infected group with RA or connective tissue disease and the matched HAM/TSP controls (*P *> 0.05 in both Wilcoxon's test and the paired *t*-test). As expected, the difference between the asymptomatic carriers and the HAM/TSP group was significant (*P *= 0.0001 in Wilcoxon's test, *P *< 0.0001 in the paired *t*-test).

**Table 1:** Clinical and biological features of HTLV-1-infected patients with RA

Patient	Sex	Age	Associated Diseases	Disease duration (year)	Sharp score	ESR (mm)	CRP (mg/l)	RF (IU/ml)	Treatment	HTLV-1 proviral load (copies / 10^6 ^PBMCs)
1	F	75	-	7	65	111	313	128	Corticosteroids, DMARDs	373,300
2	F	63	HAM/TSP	2	10	70	5	32	Corticosteroids, DMARDs	39,800
3	F	62	-	9	3	41	6	-	NSAIDs	126,500
4	F	58	-	7	46	5	3	64	Corticosteroids, DMARDs	55,000
5	F	72	-	7	109	56	16	-	NSAIDs, DMARDs	38,500
6	F	59	-	10	5	38	5	-	Corticosteroids, DMARDs	28,900
7	F	64	-	2	8	25	12	128	Corticosteroids, DMARDs	79,800
8	F	69	-	16	126	21	30	16	Corticosteroids, DMARDs	230,000
9	F	57	HAM/TSP	13	6	29	13	-	Corticosteroids	247,400
10	F	58	HAM/TSP	2	17	35	9	32	Corticosteroids, DMARDs	106,500
11	F	42	-	18	3	22	5	-	Analgesics	32,200
12	F	77	HAM/TSP	7	110	88	41	-	Corticosteroids, DMARDs	14,600

Sharp score : joint space narrowing score and erosion score; CRP: C-reactive protein; ESR, erythrocyte sedimentation rate; RF: rheumatoid factor; NSAIDs: non-steroidal anti-inflammatory drugs; DMARDs: disease-modifying anti-rheumatic drugs (hydroxychloroquine, leflunomide, methotrexate, etc.)

**Table 2:** Clinical and biological features of HTLV-1-infected patients with connective tissue disease

Patient	Sex	Age	Connective tissue disease	Associated diseases	Disease duration (year)	ESR (mm)	CRP (mg/l)	ANA	Treatment	HTLV-1 proviral load (copies / 10^6 ^PBMCs)
13	F	38	SLE	uveitis	13	38	6	+	Corticosteroids, HC	18,400
14	F	68	PM	-	1	17	1	+	Corticosteroids	411,200
15	F	60	SS	-	2	10	1	+	NSAIDs, HC	1,500
16	F	56	PM	HAM/TSP	10	17	12	+	Corticosteroids	71,700
17	F	63	SS	-	3	22	15	+	NSAIDs, HC	169,900
18	F	65	SS	HAM/TSP, alveolitis	5	21	1	-	Corticosteroids	362,300

SS, Sjögren's syndrome, SLE : Systemic lupus erythematosus, PM : Polymyositis, ANA: anti-nuclear antibodies; HC : hydroxychloroquine

Of the 12 HTLV-1-infected patients with RA, 4 (patients 2, 9, 10, and 12) had HAM/TSP (Table 1); their respective HTLV-1 proviral loads were 39,800, 247,400, 106,500, and 14,600 copies per 10^6 ^PBMCs. Two patients with connective tissue disease (Table 2, patients 16 and 18) had HAM/TSP, which was associated with alveolitis in patient 18, with proviral loads of 71,600 and 362,300. These samples did not account for the higher proviral load in the arthritic group compared to the asymptomatic carrier group. Indeed, after excluding the patients with co-existing HAM/TSP, the difference between the asymptomatic controls and the group with RA or connective tissue disease remained significant (*P *= 0.0121, Wilcoxon's test; *P *= 0.0027, paired *t*-test), even when the analysis was restricted to the subgroup with RA alone (*P *= 0.0117, Wilcoxon's test; *P *= 0.0003, paired *t*-test). The patient with connective tissue disease and uveitis (patient 13) had 18,420 copies per 10^6 ^PBMCs. For one of the patients with RA and HAM/TSP (patient 10), 4 consecutive frozen dry pellets of PBMCs from 1996 to 2002 were available; in these, the proviral load was relatively stable at 92,100, 73,600, 143,300, and 106,500 copies per 10^6 ^cells, respectively.

No correlation was found between HTLV-1 proviral load and the age of the patient, the duration of illness, or the Ritchie's index score (*P *> 0.05, Spearman test). HTLV-1 proviral load did not correlate with erythrocyte sedimentation rate or C-reactive protein level (*P *> 0.05, Spearman test). No significant difference in HTLV-1 proviral load was seen in patients positive or negative for rheumatoid factor or antinuclear antibody (*P *> 0.05, Mann-Whitney test) or receiving or not receiving either specific treatments for RA or corticotherapy (*P *> 0.05, Mann-Whitney test).

The proviral load was measured in two sets of PBMCs and synovial fluid or synovial tissue cells obtained from one HTLV-1-infected patient with RA at an interval of one year (Table 1, patient 5). In the first set of samples, the HTLV-1 proviral load in the synovial fluid cells and paired PBMCs was 845,200 and 125,300 copies per 10^6 ^cells, respectively, while, in the second set of samples from a year later, the proviral load in the synovial tissue cells and paired PBMCs was 666,700 and 38,500 per 10^6 ^cells, respectively.

**Figure 2:**
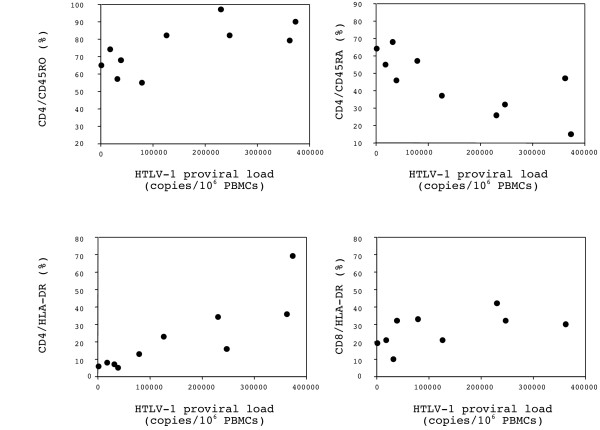
Correlation between HTLV-1 proviral load, and memory or activated T lymphocytes subsets in the peripheral blood from HTLV-1-infected patients with RA or connective tissue disease.

Lymphocytes subsets and activation status were examined in the peripheral blood of HTLV-1-infected patients with RA or connective tissue disease. When correlations were examined between HTLV-1 proviral load and the percentage of T lymphocytes expressing CD45RO, CD45RA, or HLA-DR in the HTLV-1 infected patients with RA or connective tissue disease (Figure 2), HTLV-1 proviral load correlated positively with the percentage of CD4+ T cells expressing CD45RO and negatively with that of CD4+ T cells expressing CD45RA (*P *= 0.039 and *P *= 0.021, Spearman test). A positive correlation was found between HTLV-1 proviral load and the percentage of CD4+ T cells expressing HLA-DR (*P *= 0.008), while the correlation did not reach significance for CD8+ HLA-DR T cells.

Lymphocyte distribution and activation were compared in the peripheral blood and synovial fluid of one HTLV-1-infected patient with RA (patient 5). The percentage of CD4+ T cells expressing CD45RO was dramatically increased in the synovial fluid (98%) compared to the peripheral blood (48%), as was the percentage of CD4+ T lymphocytes expressing HLA-DR (94% compared to 18%).

## Discussion

The etiology of autoimmune diseases, such as RA or mixed connective tissue disease, has yet to be established, but appears to result from complex interactions between host genetic and environmental factors [36], and retroviruses have been considered as possible causative factors [37]. HTLV-1 has been suggested to be implicated in the pathogenesis of RA in Japan, where this retrovirus is endemic [13,17,18]. Epidemiological studies have shown an association between HTLV-1 infection and RA in Japan [19,20], and between HTLV-1/2 infection and arthritis in the United States [22]. HTLV-1-seropositive cases with connective tissue disease have also been described, although the authors suggested a geographical, rather than an etiological, link [14]. Another study failed to demonstrate any association between HTLV-1 infection and systemic lupus erythematosus in Jamaica [15]. Considering the age, sex ratio, and HTLV-1 prevalence (4.6%) in our RA cohort, a fortuitous coincidence cannot be excluded. The prevalence of HTLV-1 infection in Martinique is estimated to be around 1.5% and increases with age, particularly among women [38]. An epidemiological study is therefore required to determine whether there is no, or a weak, association between HTLV-1 and arthritis in Martinique.

Nevertheless, the present study provides biological data suggesting a contribution of HTLV-1 to the development of some cases of RA or mixed connective tissue disease. We found that: (1) The circulating HTLV-1 proviral load was higher in HTLV-1-seropositive patients with RA or connective tissue disease than in asymptomatic HTLV-1 carriers and similar to that in patients with HAM/TSP; (2) In the peripheral blood of patients with arthritis, HTLV-1 proviral load correlated with the percentage of memory and activated T CD4^+ ^cells; (3) A high HTLV-1 proviral load was found in the synovial fluid and tissue cells in a patient with RA; (4) In this patient, the percentage of memory and activated CD4^+ ^T cells was higher in the synovial compartment than in the peripheral blood.

The HTLV-1 proviral load is thought to be a major determinant of HTLV-1-associated diseases. The HTLV-1 proviral load is higher in the peripheral blood from patients with HAM/TSP than in blood from asymptomatic carriers [34]. It is also higher in the peripheral blood of patients with HTLV-1-associated uveitis than in asymptomatic carriers [39,40]. Similarly, we observed a significantly higher proviral load in HTLV-1-infected patients with either RA or connective tissue disease than in HTLV-1 asymptomatic carriers. Moreover, the proviral load was higher in the synovial fluid and tissue from one HTLV-1-infected patient with RA than in the peripheral blood. Assuming an average of one HTLV-I provirus per infected cell, the proviral load values in these synovial samples suggest that the majority of infiltrated cells are infected. HTLV-1 proviral load is known to be higher in the spinal fluid than in paired blood samples from HAM/TSP [41,42], but not asymptomatic carriers [43]. Interestingly, a higher HTLV-1 proviral load than in the peripheral blood has also been reported in bronchoalveolar lavage fluid in patients with HTLV-1-associated alveolitis [44] and in the labial salivary glands in patients with HTLV-1-associated Sjögren's syndrome [45,46]. Thus, a high proviral load might be involved in the pathogenesis of several other HTLV-1-associated inflammatory disorders in addition to HAM/TSP.

In HAM/TSP, the HTLV-1 proviral load reaches an equilibrium set-point that is correlated with progression of motor disability, and fluctuates by no more than 2- to 4-fold over a decade [35]. In one HTLV-1-infected patient with RA, the proviral load was found to be stable over a 6 year period. In HTLV-1-associated uveitis, the proviral load has been shown to correlate with disease activity [40]. However, in our cohort, HTLV-1 proviral load in the peripheral blood did not correlate with disease activity and was not influenced by treatment of the rheumatological disease. This suggests that HTLV-1 proviral load reaches a set-point determining the onset of the rheumatological disease, but the intensity of the symptoms might be influenced by subsequent in situ events.

The unusually high proviral loads in HTLV-1 infection results mainly from the Tax-driven activation and expansion of infected cells [47]. The HTLV-1 targets are mainly CD45RO-expressing CD4^+ ^T lymphocytes and the proviral load is reported to correlate with the number of memory T cells [48]. In our HTLV-1-infected cohort with RA or connective tissue disease, HTLV-1 proviral load correlated positively with the percentage of CD4^+ ^T cells expressing CD45RO and negatively with that of CD4^+ ^T cells expressing CD45RA. The HTLV-1 proviral load also correlated positively with the percentage of HLA-DR-expressing T cells. Migration of HTLV-1-infected CD4^+ ^T cells and HTLV-1-specific CD8^+ ^cytotoxic T lymphocytes (CTL) into the central nervous system is a critical step in the pathogenesis of HAM/TSP [31,32]. Similarly, infiltration of T cells plays a central role in the initiation and perpetuation of RA [29,30]. Thus, our finding of an increase in memory (CD45RO) and activated (HLA-DR) CD4^+ ^T cells in the joint fluid of an HTLV-1 carrier with RA supports the hypothesis of a pathogenic involvement of HTLV-1-infected T lymphocytes.

Several mechanisms are potentially involved in the occurrence of rheumatological disorders during HTLV-1 infection. Firstly, HTLV-1 infection upregulates the expression of adhesion molecules potentially involved in the migration of lymphocytes into the spinal and joint compartments [17]. Secondly, HTLV-1 might be transmitted from the infiltrated T lymphocytes to the synoviocytes [23-26], and subsequent Tax expression might induce proliferation of these cells. Extracellular Tax protein has also been reported to stimulate the proliferation of synoviocytes [49]. Finally, Tax expression stimulates the production of a variety of cytokines, including IL-15 and its receptor. IL-15 might represent a cornerstone between HAM/TSP and HTLV-1-associated rheumatological diseases. IL-15 favors T cell migration into the target tissue compartment [50]. Moreover, IL-15 inhibits IL2-mediated activation-induced cell death, and is suspected to both facilitate the persistence of MHC I restricted memory CD8+ T cells involved in the pathogenesis of HAM/TSP, and enhance the survival of self-reactive T cells, leading to the development of autoimmune disease [51]. Recent data argue for a possible autoimmune mechanism of tissue damage in HAM/TSP [52]. Moreover, IL-15 can also induce TNF-α synthesis by macrophages, which, in turn, stimulates a cascade of proinflammatory cytokines, including IL-1β, IL-6, and GM-CSF [50,53], which induce synoviocyte proliferation and are thought to be deleterious for the central nervous system [31]. Thus, the coexistence of HAM/TSP in one-third of the HTLV-1-infected patients with RA might be explained by the shared features of a high proviral load and common downstream pathways. Alternatively, autoimmune arthritis or its etiological factors might secondarily enhance HTLV-1 proviral load through cell activation, with subsequent migration of HTLV-1-infected cells into the joint or CNS. The accumulation of HTLV-1-infected lymphocytes in the synovium could result from selective infiltration and/or from oligoclonal expansion once the PBMCs have infiltrated the joint. Whether the increase in HTLV-1-infected cells in the peripheral blood and the even greater increase in the synovial compartment are the cause or an effect of the associated arthritis remains uncertain. A role of the anti-inflammatory and anti-rheumatic drugs can also not be excluded.

In conclusion, our data in HTLV-1-infected patients with RA or connective tissue disease are consistent with a role of the proviral load in the development of these rheumatological disorders, although the direction of causality in this interaction remains open to question. HTLV-1 might cause a systemic immune-mediated inflammatory disease potentially involving tissues other than the central nervous system, HAM/TSP being only the major syndrome. The clinical expression of this disease might be determined by the amount of HTLV-1-infected T lymphocytes, their level of activation, and their capacity to accumulate in different body compartments. Further research is needed to increase our knowledge of the molecules involved in the homing of HTLV-1-infected CD4+ T lymphocytes and of anti-HTLV-1-specific CD8+ CTL to different target tissues.

## Materials and Methods

### Patients

The study was performed in Martinique, an island in the lesser Antilles archipelago, with a population of 400,000. Between 1988 and 2001, 280 patients with RA, defined according to the American Rheumatology Association (ARA) criteria, and 335 patients with connective tissue disease were followed on an inpatient or outpatient basis at the Rheumatology Department of the Regional Teaching Hospital. Thirteen (4.6%) of the 280 patients with RA (1 male and 12 female) were found to be HTLV-1 seropositive, confirmed by Western blotting (antibodies recognizing at least rgp21, p19, and p24) and peripheral blood was obtained from 12 of these. In addition, 6 patients with connective tissue disease (three with Sjögren's syndrome, two with inflammatory myopathy, and one with systemic lupus), who were seropositive for HTLV-1, were included in the study. Samples were collected between September 2001 and June 2002. For one patient, sequential samples cryopreserved since 1996 were available.

The mean age at the time of sampling was 63 years for the HTLV-1-seropositive RA patients compared to 60 years for the total RA cohort, while the mean age of the HTLV-1-seropositive patients with connective tissue disease was 58 years. The mean time interval between onset of the auto-immune disease and sampling for HTLV-1 proviral load determination were 8 years and 6 years in the patients with RA and connective tissue disease, respectively. Of the 12 HTLV-1-seropositive RA patients, 4 had HAM/TSP, defined according to the WHO guidelines [54]. Two of the patients with connective tissue disease also presented HAM/TSP. The clinical and biological features of the HTLV-1-infected patients with either RA or connective tissue disease are summarized in Tables 1 and 2, respectively.

Each HTLV-1-infected patient with RA or connective tissue disease was matched for age (± 5 years) and sex with 2 patients with HAM/TSP and 2 asymptomatic HTLV-1 carriers.

### Measurement of HTLV-1 proviral load

PBMCs were isolated from EDTA blood by density gradient centrifugation. Synovial fluid samples were obtained by arthrocentesis and synovial tissue was obtained during arthroscopy. The synovial tissue was minced into small pieces, washed with phosphate-buffered saline, and passed through a wire mesh to collect synovial tissue cells. Cells were cryopreserved until use.

DNA was extracted from 10^6 ^cells using a phenol/chloroform procedure. The HTLV-1 proviral load was quantified using a real-time TaqMan PCR method [55]. SK110/SK111 primers were used to amplify a 186 bp fragment of the *pol *gene and the dual-labeled TaqMan probe (5' FAM and 3' TAMRA) was located at 4829–4858 bp of the HTLV-1 reference sequence (HTLV_ATK_). Albumin DNA was quantified in parallel to determine the input cell number and was used as an endogenous reference to normalize variations due to differences in the PBMC count or DNA extraction. For both HTLV-1 and the albumin gene, amplifications were performed on 10 μl of DNA extract using the TaqMan PCR Core Reagent kit, data being acquired with the ABI Prism 7700 Sequence Detector System (Perkin Elmer, Foster City, California, USA). Standard curves were generated using ten-fold serial dilutions of a double standard plasmid (pcHTLV-ALB) containing one copy of the target regions of both the HTLV-1 *pol *gene and the cellular albumin gene. The HTLV-1-infected human lymphocyte line MT2 (ECACC 93121518) was used as a control for quantification, the limit for an acceptable result being taken as 2.4–3.3 copies of the HTLV-1 pol gene per cell and the variation between series being normalized on the basis of three copies per MT2 cell. All standard dilutions and control and patient samples were run in duplicate for both HTLV-1 and albumin DNA quantification. Standard curves for HTLV-1 and albumin were accepted when the slope was between -3.322 and -3.743 (corresponding to amplification efficiencies of 100% to 85%) and the correlation coefficient, *r*^2^, was >0.992. If the variation between duplicate values of HTLV-1 or albumin DNA copy numbers was greater than 30%, the analysis was repeated. The normalized value for the HTLV-1 proviral load was reported as the HTLV-1 average copy number/albumin average copy number ratio × 10^6 ^and expressed as the number of HTLV-1 copies per 10^6 ^PBMCs.

### Determination of CD4- and CD8-positive lymphocyte counts

Lymphocyte subsets in PBMCs were characterized using a panel of labeled anti-human monoclonal antibodies, consisting of fluorescein isothiocyanate-conjugated anti-CD3 (clone SK7, mouse IgG_1_), allophycocyanin-conjugated anti-CD4 (clone SK3, mouse IgG_1_), peridinin chlorophyll protein-conjugated anti-CD8 (clone SK1, mouse IgG_1_), phycoerythrin (PE)-conjugated anti-CD45RO (clone UCHL-1, mouse IgG_2a_), and PE-conjugated anti-HLA-DR (clone L243, mouse IgG_2a_) (all from BD Biosciences Immunocytometry Systems, San Jose, CA), and PE-conjugated anti-CD45RA (Hl100 mouse IgG_2b_, from BD Biosciences Pharmingen). The analyses were performed on a FACSCalibur (BD Biosciences Immunocytometry Systems).

### Statistical analysis

Mann-Whitney's *U *test, Wilcoxon's signed rank test, paired Student's *t*-test, and Spearman's rank correlation were used, as appropriate. A *P *value < 0.05 was considered to be statistically significant.

## List of Abbreviations

HTLV, human T-lymphotropic virus; HAM/TSP, HTLV-1-associated myelopathy/tropical spastic paraparesis; RA, rheumatoid arthritis; PBMCs, peripheral blood mononuclear cells.

## Competing Interests

The author(s) declare that they have no competing interests.

## Authors' Contributions

MY carried out most of the clinical and experimental work. AL was involved in the molecular biology work and contributed to the design of the study. FD and GL performed the flow cytometry analysis. SO and GJB participated in the neurological and rheumatologic evaluations, respectively. SA supervised the design and the course of the clinical study. RC conceived the study and drafted the manuscript. All authors read and approved the final manuscript.
